# Airborne SARS-CoV-2 Detection by ddPCR in Adequately Ventilated Hospital Corridors

**DOI:** 10.3390/toxics13070583

**Published:** 2025-07-12

**Authors:** Joan Truyols-Vives, Marta González-López, Antoni Colom-Fernández, Alexander Einschütz-López, Ernest Sala-Llinàs, Antonio Doménech-Sánchez, Herme García-Baldoví, Josep Mercader-Barceló

**Affiliations:** 1 Molecular Biology, Health Geography, and One Health Research Group (MolONE), University of the Balearic Islands, 07122 Palma, Spain; joan.truyols@uib.cat (J.T.-V.);; 2 Institute of Biophysics, Department of Natural Sciences and Sustainable Resources, University of Natural Resources and Life Sciences, 1180 Vienna, Austria; 3 iRespire Research Group, Health Research Institute of the Balearic Islands, 07020 Palma, Spain; 4 Centre of Biomedical Research Network in Respiratory Diseases (CIBERES), 28029 Madrid, Spain; 5 Department of Chemistry, Universitat Politècnica de València, 46022 València, Spain

**Keywords:** SARS-CoV-2, airborne transmission, ddPCR, bioaerosol, carbon dioxide

## Abstract

Indoors, the infection risk of diseases transmitted through the airborne route is estimated from indoor carbon dioxide (CO_2_) levels. However, the approaches to assess this risk do not account for the airborne concentration of pathogens, among other limitations. In this study, we analyzed the relationship between airborne SARS-CoV-2 levels and environmental parameters. Bioaerosols were sampled (*n* = 40) in hospital corridors of two wards differing in the COVID-19 severity of the admitted patients. SARS-CoV-2 levels were quantified using droplet digital PCR. SARS-CoV-2 was detected in 60% of the total air samples. The ward where the mildly ill patients were admitted had a higher occupancy, transit of people in the corridor, and CO_2_ levels, but there were no significant differences in SARS-CoV-2 detection between wards. The mean CO_2_ concentration in the positive samples was 569 ± 35.6 ppm. Considering all samples, the CO_2_ levels in the corridor were positively correlated with patient door openings but inversely correlated with SARS-CoV-2 levels. In conclusion, airborne SARS-CoV-2 can be detected indoors with optimal ventilation, and its levels do not scale with CO_2_ concentration in hospital corridors. Therefore, CO_2_ assessment should not be interpreted as a surrogate of airborne viral presence in all indoor spaces.

## 1. Introduction

The occurrence of diseases transmitted through the airborne route has a significant medical, economic, and social impact every year. Airborne diseases are a matter of public health concern, posing a risk of a global health crisis for some pathogens. Healthcare workers are an at-risk population. The WHO estimated that 116,000 lost their lives to COVID-19 between January 2020 and May 2021 [1]. The application of measures to prevent infections is a policy that must be promoted, as the benefits would be enormous. As an example, it has been estimated that the prevention costs for 10 years would be only about 2% of the costs of the COVID-19 pandemic [2]. The development and the improvement of preventive strategies is, therefore, a priority strategic research line in the field of airborne diseases. The spread of airborne diseases occurs predominantly in indoor settings. A major challenge in controlling indoor infection risk is the assessment of the airborne load of the infection-causing microbes. The quantification of airborne microbes can be approached with different procedures that entail the collection of air samples followed by the utilization of microbiological or biochemical methods. These procedures are also useful for analyzing how external factors affect microbial load [3], although they do not allow the real-time quantification of airborne microbes. In the absence of the use of applications for the direct real-time monitoring of airborne microbes, indicators of ventilation are used instead, such as the real-time measurement of carbon dioxide (CO_2_) levels [4,5,6,7]. Corrective actions to reduce infection risk are implemented according to this surrogate [8,9].

Mathematical models of airborne infection have been developed based on CO_2_ assessment, including the Wells–Riley equation that uses the outdoor air supply rate and the Rudnick and Milton model, which considers CO_2_ as a surrogate of exhaled breath [10]. These models use the basic reproductive number (i.e., the expected number of secondary infections that arise from a single infectious case where all individuals are susceptible) and are of great value to estimate indoor airborne infection risk [11]. According to these theoretical models, the CO_2_ threshold levels above which the infection risk increases have been established for SARS-CoV-2 [7,12], and recommendations on CO_2_ levels are issued for many organizations [13,14]. However, these models have limitations in their applicability because they do not account for the concentration of pathogens in the air, among other influencing factors, such as respiratory activity and type of pathogen. An infective microbial load might occur under relatively low CO_2_ levels, and, oppositely, high CO_2_ levels do not necessarily correlate with the load of infective airborne microbes. Indeed, several organizations do not recommend CO_2_ concentration as a metric of infection risk [15]. Therefore, it is essential to develop strategies to quantify airborne microbes in indoor air, as well as to analyze the relationship between microbial load and measurable environmental factors to improve both the estimation of infection risk and the understanding of influencing factors.

The presence of airborne pathogens has been identified in indoor air samples, especially for SARS-CoV-2 [16,17,18]. Moreover, the relationship between the presence of airborne SARS-CoV-2 and CO_2_ concentration has been investigated with mixed results. While the airborne viral load has been positively correlated with CO_2_ concentration in some studies [19], others found no correlation [20,21]. Two critical issues for the SARS-CoV-2 quantification in air samples are the efficient collection of viral-laden bioaerosols and the sensitivity of the detection method. We developed a protocol for air samples that combined a high efficiency in bioaerosol collection with a high sensitivity in the SARS-CoV-2 genome detection [22]. In the present study, we used this protocol to analyze the influence of environmental factors on airborne SARS-CoV-2 genome detection in a hospital. Airborne SARS-CoV-2 was detected in hospital corridors at relatively low CO_2_ levels, indicating that CO_2_ concentration cannot always be used as an indicator of the presence of SARS-CoV-2.

## 2. Materials and Methods

### 2.1. Sampling Points

Air sample collection was carried out in two hospital wards (2N and 3O) designated for COVID-19 patients at the Hospital Universitari Son Espases (HUSE) in Palma de Mallorca, Spain, during January and February 2022. Ward 2N housed patients with milder forms of COVID-19, who required minimal medical care and generally did not need oxygen or only required it at low concentrations. In contrast, ward 3O functioned as an Intermediate Respiratory Care Unit (IRCU), admitting patients with more significant respiratory compromise who were receiving high-concentration oxygen therapy and, in some cases, required high-flow oxygen therapy (HFOT) (between 30 and 60 L/min, respectively).

**Figure 1:**
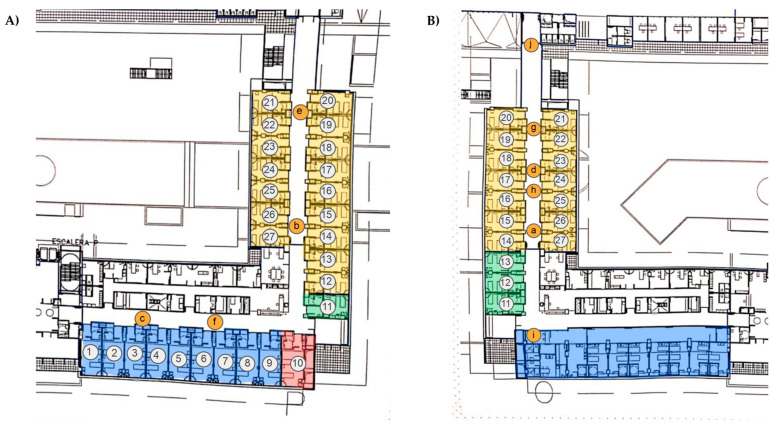
Plan of the hospital wards. Bioaerosol samples were collected in the corridors of the wards 2N (**A**) and 3O (**B**) at the indicated sampling points (from a–j). COVID-19 patient rooms are numbered. Colors are used to show the single rooms (yellow), double rooms (blue), negative pressure rooms (green), and the room used for medical staff to change clothing (red).

The layout of ward 2N is L-shaped and includes a mix of double-occupancy rooms and a dedicated room for healthcare staff to change clothing (Figure 1A). Meanwhile, ward 3O has a rectangular configuration with only single-occupancy rooms, three of which are equipped with negative pressure systems (Figure 1B).

### 2.2. Bioaerosol Sample Collection

The collection system used was the BioSampler^®^ (SKC Inc., Valley View, Eighty Four, PA, USA). This liquid impinger was used in previous studies by our group [3,17,22]. This device allows the collection of aerosols of 1–3 µm with a high efficiency [23], presents a high performance in collecting RNA viruses [24], and has a high rate of virus preservation [25]. Bioaerosols were collected in a sterile vessel with 1 mL of Viatrap^®^ (SKC, Valley View, Eighty Four, PA, USA) mineral oil, which allows the retention of SARS-CoV-2-laden bioaerosols [22]. The sampling device was placed at a 1.3 m height at the locations indicated in Figure 1. A total of forty samples were carried out on ten different days. Sixteen air samples were collected in ward 2N and twenty-four in ward 3O. On each sampling day, four consecutive 1 h samples were taken at the same sampling point.

### 2.3. RNA Isolation and Quantification

RNA isolation from air samples was performed by using the phenol extraction method [17]. Briefly, the collection media were immediately placed on ice and manipulated in a laboratory with a class II safety cabinet. Three aliquots were transferred into sterile microcentrifuge tubes. Then, 750 µL of TRItidy G™ (Panreac AppliChem, Barcelona, Spain) were added to each tube, and the protocol detailed in [17] was followed. The RNA aliquots from air samples were unified, and the total RNA was quantified with a Synergy H1 spectrophotometer. Afterwards, the RNA samples were stored at −80 °C.

### 2.4. Reverse Transcription

Complementary DNA (cDNA) was synthesized using the Superscript II enzyme (Invitrogen, Waltham, MA, USA), following the protocol described in [22], with which a single SARS-CoV-2 RNA can be detected. For each reaction, 5 µL of total RNA was combined with 1 µL of random nonamers and 1 µL of a 10 mM deoxynucleotide triphosphate (dNTP) mix, in a final volume of 12 µL. The mixture was incubated at 65 °C for 5 min and then rapidly chilled on ice. Subsequently, 4 µL of 5× First-Strand Buffer (Invitrogen, Waltham, MA, USA), 2 µL of 0.1 M dithiothreitol (DTT) (Invitrogen), and 1 µL of Riboprotect RNase inhibitor (40 U/µL) were added. The reaction was then incubated at 25 °C for 2 min. Finally, 1 µL (200 units) of Superscript II reverse transcriptase was added. The complete reaction was incubated at 25 °C for 10 min, 42 °C for 50 min, and 70 °C for 15 min. Negative controls were included. cDNA samples were kept at −20 °C.

### 2.5. Droplet Digital Polymerase Chain Reaction (ddPCR)

ddPCR was selected for airborne SARS-CoV-2 genome quantification, as we previously estimated that the limit of detection is 1.1 copies [17]. Reactions were prepared from 6 µL of non-diluted cDNA, and the PCR protocol was run as described in [17]. Briefly, cDNA was mixed with 10 μL of ddPCR Supermix for Probes (no dUTP) (Bio-Rad, Hercules, CA, USA), 1 μL of each forward and reverse primer solution at 18 μmol/L, and 1 μL of 5 μmol/L FAM- and HEX-labeled probe solution, in a final volume of 20 μL. A QX200™ Droplet Generation Oil (Bio-Rad, Hercules, CA, USA) was used to generate oil droplets with 20 μL of ddPCR solution mix and 70 μL of QX200™ Droplet Generation Oil (Bio-Rad) charged into DG8™ Cartridges (Bio-Rad). Next, 40 μL of the emulsion was transferred into a 96-well PCR plate. Forward and reverse primers and probe sequences were 5′-CCCTGTGGGTTTTACACTTAA-3′, 5′-ACGATTGTGCATCAGCTGA-3′ and 5′-HEX-CCGTCTGCGGTATGTGGAAAGGTTATGG-TAMRA-3ʹ for the ORF1ab. Positive and negative controls were included in each run.

### 2.6. Environmental Data

Environmental temperature (°C), relative humidity (% RH), and CO_2_ concentration (ppm) were obtained with a C-LOGIC 7100-AQ air quality monitor (MGL EUMAN S.L., Argame-Morcín, Spain), which was placed next to the BioSampler^®^ device. The measurement range and accuracy of the device were as follows: 400–5000 ppm and ±50 ppm, for CO_2_; −10 °C to +50 °C and ±1 °C for temperature; and 20–85% and ±4% for relative humidity. The resolution of the non-dispersive infrared sensor for CO_2_ is 1 ppm. Environmental data were collected in parallel to the 1 h air sampling and recorded every five minutes.

To estimate the degree of air communication between the patient rooms and the corridor, we recorded the number of times that the positive pressure COVID-19 patient room doors located in the corridor where the BioSampler^®^ was placed were opened. The counts within a 5 min period were recorded over the 1 h air sampling. To analyze the relationship between CO_2_ and occupancy, we collected the number of occupied COVID-19 rooms, as well as the number of people present in the corridors, which was recorded within a 5 min period over the 1 h air samplings.

### 2.7. Statistical Analysis

We assessed normality using the Shapiro–Wilk test (α = 0.01) and inspected Q–Q plots. We compared normally distributed continuous variables with Student’s t-test and non-normal variables using the Kruskal–Wallis test. We analyzed categorical variables with the chi-square test or, when any expected cell count was <5, Fisher’s exact test. For correlations, we computed Pearson coefficients for pairs of normally distributed variables and Spearman coefficients otherwise. All analyses were performed in R Studio (version 4.2.0; R Foundation for Statistical Computing, Vienna, Austria).

## 3. Results and Discussion

### 3.1. Airborne SARS-CoV-2 Detection in Hospital Corridors

A total of 40 air samplings were carried out in two corridors of HUSE over ten different days, across ten distinct locations, and in parallel with the registration of the environmental parameters. The data of each sampling was compiled in Table 1, detailing the mean value of the temperature (°C), relative humidity (% RH), CO_2_ concentration (ppm), people count, openings of COVID-19 patient room doors, and the estimated airborne viral concentration (SARS-CoV-2 genome copies per m^3^) detected by ddPCR.

Sixty percent of the samples tested positive for SARS-CoV-2, with viral loads ranging from 10.67 to 53.33 copies per m^3^. The robustness of these results is supported by the selection of the methodology and the subsequent optimization to improve the efficiency of SARS-CoV-2 collection and the sensitivity to detect the viral genome [22]. Firstly, the use of the BioSampler for air sampling allows the collection of particles ranging from 1 to 3 μm with an efficiency of 97%, surpassing that of 28 other commercially available bioaerosol collection devices [23]. Moreover, this device had greater virus preservation [25] and RNA recovery efficiency from the *Influenza* virus compared to other samplers [24]. The use of mineral oil enabled extended sampling durations due to its low volatility; additionally, as reaerosolization is minimized, overall collection efficiency results improved [22]. Regarding sensitivity, we previously assessed that the lowest measured ORF1ab target number was a single RNA copy by using the Superscript II reverse transcriptase and ddPCR [22]. The estimated limit of detection using ddPCR was 1.1 SARS-CoV-2 cDNA copies per reaction [17]. In addition, ddPCR allows a more accurate and reproducible quantification of low-abundance targets than qPCR [26]. Its application is suitable for the quantitative assessment of SARS-CoV-2 levels in air [27] and biological [28] samples, in which the target concentration is very low. This means that the number of SARS-CoV-2 genomic copies obtained in the present study is highly reliable despite being low values. Altogether, these capabilities further support the robustness of the results obtained with this protocol.

On the other hand, one potential limitation we faced was the relatively low airflow rate of the BioSampler^®^ (12.5 L/min). Although there are numerous samplers available with higher flow rates, reaching up to 1000 L/min [23], we selected this sampler due to the above-mentioned advantages. To increase the total volume of collected air, four consecutive samplings were carried out, thereby collecting 3.000 L in each sampling point. SARS-CoV-2 was detected in all sampling points, except in point c of ward 2N (Figure 1A), indicating that its presence is widely distributed across the corridors. At the sampling point “c”, there was no viral load, probably because this point is located at one end of the corridor where there are only patient rooms on one side. Our procedure and all the other procedures used to assess the airborne SARS-CoV-2 levels have their strengths and weaknesses, and it is necessary to standardize a method for monitoring the airborne viral load under diverse indoor conditions to minimize infection risk [29,30].

Another limitation of the present study is the absence of infectivity assays to evaluate whether the collected bioaerosols contained viable SARS-CoV-2 particles; therefore, the public health implications are limited. However, previous studies have demonstrated that viable and potentially infectious viral particles persist in air samples [31], even at low RNA concentrations [32,33], and at relatively large distances from the emitter [34] and time after dispersion [35]. This evidence contributed to the claim that airborne transmission is the dominant route of SARS-CoV-2 infection [36]. Our results reinforce the need to use personal protective measures in these areas.

In our previous study carried out inside COVID-19 patient rooms, SARS-CoV-2 was detected in 53.6% of the air samples [3]. This result closely aligns with the findings of the present study and is consistent with other reports that have also identified the presence of SARS-CoV-2 in hospital corridors [37,38]. Since there is no other likely source than the rooms, the virus could be transported from there to the corridor airflow as it has been described [31,34,35].

### 3.2. Comparison of the Environmental Data Between Hospital Wards

Ward 2N has a greater capacity for admitting patients (Figure 1) and showed a significantly greater occupancy during the sampling days and, consequently, a higher door-opening frequency than ward 3O (Table 2). In contrast, the number of individuals present in the corridors did not significantly differ between the wards. Although ward 3O shows a lower patient occupancy and door-opening frequency, this did not translate into a lower number of personnel present in the corridor, since the admitted patients required higher care demands than those admitted in ward 2N.

Mean temperature values ranged from 23.7 °C to 27 °C in ward 2N and from 22.4 °C to 26 °C in ward 3O, while mean relative humidity values ranged from 21.1% to 27.8% in ward 2N and from 17.6% to 33.2% in ward 3O, with no significant differences in both parameters between the two wards. These environmental conditions fall within the recommended indoor temperature and humidity range [39,40]. In contrast, CO_2_ levels were significantly higher in ward 2N (Table 2). The readings ranged from 453 to 832 in ward 2N and, from 447 to 684 in ward 3O. The CO_2_ threshold to establish indoor air quality according to the Spanish regulation is 500 ppm above the outdoor CO_2_ levels [40,41]. Assuming an external CO_2_ concentration of 400 ppm, none of the readings surpassed 900 ppm in our study, indicating that the corridors were adequately ventilated.

Although the samplings were conducted in areas without patients and where everyone was wearing masks, airborne SARS-CoV-2 was detected in both corridors. Despite all the sampling points from ward 3O resulting positive in at least two samplings, both the proportion of positive samples and the viral load quantification were not significantly different between corridors (Table 2). A higher viral load could have been expected in the ward where the more critical patients are admitted (3O) because in a previous study performed in patient rooms, it was found that the severity of COVID-19 disease is associated with a higher SARS-CoV-2 detection [3]. However, the higher occupancy in ward 2N probably counteracted this expectation. Finally, the relatively low number of samples collected in each ward may have precluded obtaining significant differences in the airborne SARS-CoV-2 detection or quantification between corridors.

### 3.3. Relationship Between Airborne SARS-CoV-2 Detection and Environmental Parameters

An analysis was conducted to assess whether environmental variables were associated with SARS-CoV-2 detection in the air samples. The use of a highly sensitive method for the airborne SARS-CoV-2 detection ensures the reliability of the results of this analysis, displayed in Table 3. The order of the samplings performed at the same sampling point was not related to SARS-CoV-2 detection. No significant differences between positive and negative airborne SARS-CoV-2 samples were detected regarding occupancy level, frequency of COVID-19 patient room door openings, or the number of people in the corridor (Table 3). With regard to the air parameters, no significant differences were observed in temperature or relative humidity values between SARS-CoV-2-positive and -negative samples.

However, a statistically significant difference in CO_2_ concentration was found between samples with and without viral presence. CO_2_ concentration was higher in negative samples compared to the positive ones (Table 3). This finding contrasts with the assumption that CO_2_ concentration and SARS-CoV-2 are positively correlated. Based on CO_2_ assessment, the concentration thresholds above which the infection probability increases have been estimated in several conditions. Thus, Iwamura et al. [12] determined that CO_2_ levels should remain below 654 ppm for inpatient settings or 854 ppm for outpatient settings to ensure an infection probability below 1% when N95 masks are used. However, in our study, SARS-CoV-2 was detected below 654 ppm, meaning that there is a potential infection risk at CO_2_ concentrations that might be widely considered as secure. The risk of SARS-CoV-2 infection estimated by CO_2_ concentrations largely varies depending on the space [7]. In our study, the hospital corridors where bioaerosols were sampled were relatively large spaces, equipped with an air extraction device every four rooms, and characterized by the intermittent presence of people at a relatively low density. Our findings support the ASHRAE’s position in the sense that CO_2_ concentration does not capture the impacts of reduced occupancy, particle filtration, or air cleaning [15].

### 3.4. Relationship Between Airborne SARS-CoV-2 Quantification and Environmental Parameters

**Figure 2:**
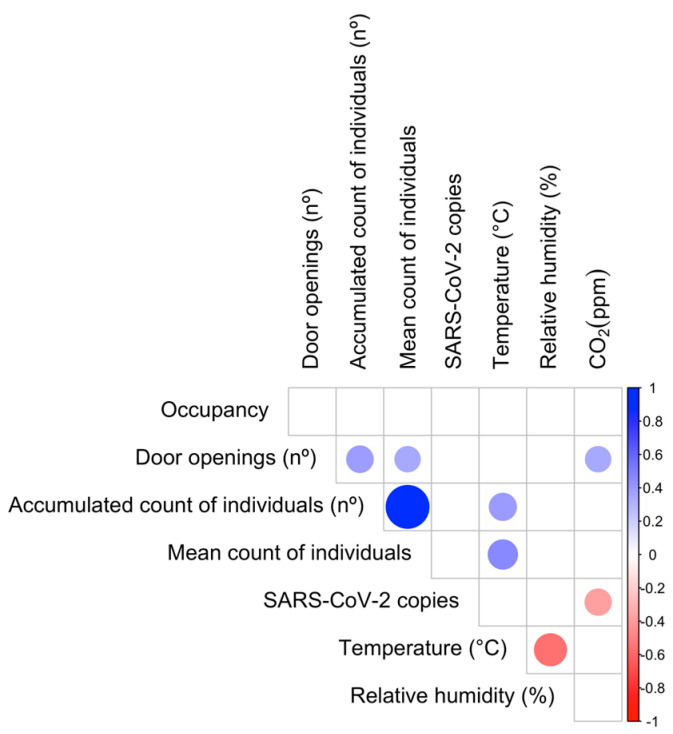
Correlation analysis between environmental parameters. Circles indicate statistically significant correlations. Blue and red circles indicate positive and negative correlations, respectively. Pearson or Spearman tests were conducted.

The next approach was to analyze the correlations between the environmental variables (Figure 2). Expected correlations were found, such as the inverse correlation between temperature and relative humidity. Both variables affect SARS-CoV-2-laden bioaerosols and infectivity [42]. Intermediate indoor relative humidity values (40–60%) were associated with a lower spread and severity of COVID-19 outbreaks compared to extreme values [43], while differences in temperature (from 10 to 40 °C) have a greater influence than relative humidity on SARS-CoV-2 decay [44]. In our study, SARS-CoV-2 levels were not associated with relative humidity or temperature, probably because these variables were maintained within a narrow range. Another expected correlation was between the counting of people and door openings with temperature, as the intensity of human activity significantly influences indoor thermal conditions [45]. Moreover, the opening of patient doors was also positively correlated with the CO_2_ levels assessed in the corridor, suggesting there is airflow from the patient rooms, which might be less ventilated, to the corridors.

Finally, a negative correlation was found between CO_2_ levels and the copies of the SARS-CoV-2 genome. Notably, these findings contrast with previous studies that have reported a positive correlation between CO_2_ concentration and viral presence [19] and with its stability [46], which supports the use of CO_2_ levels as a surrogate of infection risk. Monitoring CO_2_ levels has been extensively described in the literature as an environmental indicator of the potential presence of airborne viruses in both hospital and non-hospital settings, and various control measures have been implemented in indoor environments to manage these levels [8,9,47]. However, while CO_2_ levels could parallel airborne viral load in many cases, they do not scale with SARS-CoV-2 load in several conditions, as in the case of a low occupancy level, as it occurs in our study. This might be due to differences in the production rates of CO_2_ and SARS-CoV-2-laden respiratory aerosols, which are conditioned by the patient viral load and by the ratio of infected to non-infected individuals. Likewise, differences in the removal rates of CO_2_ and bioaerosols will also influence the relationship between CO_2_ and SARS-CoV-2 levels, since despite both rates depending on ventilation, the removal rate of bioaerosols also depends on deposition rate and filter efficiency [48,49,50]. Thus, while it has been demonstrated that there is a linear increase between both CO_2_ and respiratory aerosols for 10 min [51], this correlation could disappear thereafter because of several factors.

Another situation in which airborne microbes can be present in adequately ventilated indoors is when demand-controlled ventilation systems using CO_2_ are used [15]. These systems will not be activated during low occupancy, thereby increasing the airborne microbial load and potentially increasing infection risk. Thus, while certain public health authorities, such as REHVA and the CDC, have issued guidelines or even regulations regarding indoor CO_2_ concentrations to mitigate airborne disease transmission, others, such as ASHRAE, do not endorse specific CO_2_ thresholds as reliable metrics for infection risk or ventilation adequacy [15]. Our work provides data that supports this recommendation.

## 4. Conclusions

The airborne SARS-CoV-2 genome was detected in the hospital corridors of two wards that differed in the severity of COVID-19 among the housed patients. SARS-CoV-2 levels in the ward corridors were not different. The SARS-CoV-2 genome was detected in 60% of the air samples within a range of 10.7 to 53.3 copies/m^3^. These results reinforce the need of individual protection measures in these areas. Bioaerosols were collected using a highly efficient sampler, and the SARS-CoV-2 genome was quantified by ddPCR, a method with high sensitivity and accuracy. However, the lack of standardized protocol to assess the levels of airborne microbes represents a limitation of this study. The viability of SARS-CoV-2 particles was not evaluated, limiting public health implications. The airborne SARS-CoV-2 genome was detected in corridors with optimal ventilation, assessed by the CO_2_ measurement. Remarkably, SARS-CoV-2 levels were not concomitant with CO_2_ concentrations. Thus, CO_2_ assessment should not be interpreted as a surrogate of airborne viral presence in hospital corridors. Our results stress the need to increase knowledge in the airborne transmission of infectious diseases, and particularly in the development of strategies aimed at monitoring the airborne concentration of microorganisms.

## Figures and Tables

**Table 1:** Environmental data collected in each sampling.

Sample	Date	Ward	Sampling Point	Occupied/Total Rooms	People Counting Mean	Door Openings	Temperature (°C)	Relative Humidity (%)	CO_2_ (ppm)	SARS-CoV-2 Copies/m^3^
1	25 Jan 2022	3O	A	6/17	3.6 ± 1.6	27	24 ± 1.4	30.5 ± 2.7	596.7 ± 39.6	12.4
2					3.1 ± 1.4	14	25.8 ± 0.4	27.7 ± 0.5	625.2 ± 39.7	14.2
3					4.3 ± 1.4	23	25.9 ± 0.3	27.6 ± 0.5	578.4 ± 21.6	0
4					2.8 ± 1.1	29	26 ± 0.0	26.8 ± 0.5	575 ± 19.7	0
5	26 Jan 2022	2N	B	25/26	4 ± 1.9	41	25.2 ± 1.0	26.9 ± 1.7	604.1 ± 25.8	0
6					2.7 ± 1.6	36	26.8 ± 0.4	24.9 ± 0.5	605.6 ± 20.0	0
7					4.5 ± 1.3	64	25.3 ± 0.5	27.3 ± 0.5	602.3 ± 24.8	0
8					4 ± 1.8	36	26.8 ± 0.4	26.1 ± 0.9	651.2 ± 28.6	12.4
9	27 Jan 2022	2N	C	25/26	2.9 ± 1	87	23.8 ± 0.9	26.7 ± 1.4	671.5 ± 23.1	0
10					3.5 ± 1.6	72	25 ± 0.0	26.2 ± 0.4	739.7 ± 49.1	0
11					1.7 ± 1.1	21	25 ± 0.0	25.3 ± 0.5	636.5 ± 12.0	0
12					2.3 ± 0.9	65	25 ± 0.0	25.5 ± 0.7	650.8 ± 18.2	0
13	31 Jan 2022	3O	D	7/17	3.1 ± 1.1	69	23.7 ± 0.9	27.7 ± 1.49	548.6 ± 13.6	23.1
14					2.3 ± 1.2	41	23.6 ± 0.5	23.2 ± 1.2	474.6 ±26.4	10.7
15					2.3 ± 0.6	35	24.9 ± 0.9	19.3 ± 1.2	520.1 ± 29.9	0
16					2.8 ± 0.8	28	26 ± 0.0	17.6 ± 0.5	587.3 ± 27.9	10.7
17	1 Feb 2022	2N	E	25/26	3 ± 1.4	200	23.7 ± 0.9	30.2 ± 1.2	540.3 ± 41.3	44.4
18					1.7 ± 0.5	39	25 ± 0.0	27.7 ± 0.5	562.2 ± 10.6	12.4
19					2.7 ± 1.1	44	25.2 ± 0.4	27.4 ± 0.5	563.2 ± 3.0	0
20					2.1 ± 0.8	32	24.6 ± 0.5	27.8 ± 0.5	551.3 ± 9.7	51.6
21	2 Feb 2022	2N	F	26/26	2.7 ± 1.3	43	24.4 ± 0.9	27.5 ± 1.7	611.8 ± 30.5	16
22					2.6 ± 0.7	51	25.8 ± 0.4	24.8 ± 0.6	599 ± 41.9	51.6
23					3.1 ± 0.7	36	27 ± 0.9	21.1 ± 1.4	547 ± 54.9	14.2
24					2.7 ± 0.7	19	25.42 ± 0.7	21.8 ± 0.6	523.6 ± 55.4	16
25	3 Feb 2022	3O	G	8/17	1.2 ± 0.6	28	23.4 ± 0.8	25.5 ± 1.3	569 ± 19.5	48
26					1.1 ± 0.3	14	25 ± 0.0	24.8 ± 0.8	571.7 ± 24.2	32
27					1.9 ± 1.4	9	25 ± 0.0	27.5 ± 0.5	582.5 ± 26.3	0
28					1.8 ± 0.8	28	25.7 ± 0.5	27.9 ± 0.3	590.6 ± 14.5	0
29	7 Feb 2022	3O	H	12/17	3.2 ± 0.8	57	24.2 ± 0.8	29.8 ± 1.2	592.8 ± 44.0	0
30					2 ± 0.6	23	25 ± 0.0	28.6 ± 0.7	587.7 ± 27.1	0
31					3.4 ± 1.2	65	25.2 ± 0.4	28 ± 0.0	588.1 ± 35.8	12.4
32					2.8 ± 1.8	58	25.7 ± 0.5	27.5 ± 0.5	627.1 ± 47.4	14.2
33	8 Feb 2022	3O	I	12/17	3.5 ± 1	1	24.2 ± 0.7	28 ± 1.5	512.3 ± 24.6	39.1
34					3.4 ± 1.3	6	25.5 ± 0.5	25.6 ± 0.5	542.5 ± 11.1	23.1
35					3.5 ± 1	14	25.7 ± 0.5	25.1 ± 0.3	544.8 ± 23.5	12.4
36					3.1 ± 1.6	0	25.3 ± 0.5	24.3 ± 0.5	557 ± 39.7	53.3
37	9 Feb 2022	3O	J	17/17	1 ± 0.0	18	22.4 ± 0.7	33.2 ± 0.4	539.9 ± 20.4	30.2
38					1 ± 0.0	13	23 ± 0.0	31.9 ± 0.3	547.8 ± 10.2	0
39					1.1 ± 0.3	9	23.4 ± 0.5	31.3 ± 0.5	611.6 ± 11.7	16
40					1 ± 0.0	14	24 ± 0.0	31 ± 0.4	576.6 ± 36.6	28.4

Airborne SARS-CoV-2 copies were detected by ddPCR using primers for the ORF1ab region. Door openings are the accumulated counts in the 1 h sampling period. Mean and standard deviation values for temperature, relative humidity, CO_2_ concentration, and people count were calculated from the data recorded within 1 h.

**Table 2:** Environmental data from each ward.

	[All] N = 40	2N N = 16	3O N = 24	*p*-Overall
Occupancy	14.5 [8.00; 25.0]	25.0 [25.0; 25.2]	10.0 [7.00; 12.0]	<0.001 ***
Door openings (nº)	30.5 [14.0; 45.8]	42.0 [36.0; 64.2]	23.0 [3.25; 30.5]	0.001 **
Total count of individuals (nº)	32.2 (11.8)	35.2 (10.2)	30.2 (12.5)	0.172
Mean count of individuals	2.62 (0.94)	2.87 (0.81)	2.46 (1.00)	0.166
SARS-CoV-2 detection				0.469
NO	16 (40.0%)	8 (50.0%)	8 (33.3%)	
YES	24 (60.0%)	8 (50.0%)	16 (66.7%)	
SARS-CoV-2 copies/ m^3^	12.44 [0.00; 23.11]	6.22 [0.00; 16]	12.44 [0.00; 24.44]	0.493
Temperature (°C)	24.9 (1.06)	25.2 (0.98)	24.7 (1.06)	0.094
Relative humidity (%)	26.7 (3.17)	26.1 (2.26)	27.1 (3.64)	0.281
CO_2_ (ppm)	583 (47.9)	604 (56.9)	569 (35.6)	0.038 *

Airborne SARS-CoV-2 copies were detected by ddPCR using primers for the ORF1ab region. Door openings and the total number of individuals present in the corridor are the accumulated counts in the 1 h sampling period. Mean values for temperature, relative humidity, CO_2_ concentration, and people counting were calculated from the data recorded within 1 h. Continuous variables with approximately normal distributions are presented as means ± standard deviations, while variables with skewed distributions are presented as medians (first and third quartiles). ***: *p* < 0.001; **: *p* < 0.01; *: *p* < 0.05.

**Table 3:** Environmental data according to SARS-CoV-2 detection.

	[All] N = 40	NO N = 16	YES N = 24	*p*-Overall
Occupancy	14.5 [8.00; 25.0]	21.0 [8.00; 25.0]	12.0 [8.00; 25.0]	0.91
Door openings (nº)	30.5 [14.0; 45.8]	35.5 [23.0; 58.8]	28.0 [7.00; 41.5]	0.203
Count of individuals (nº)	32.2 (11.8)	33.1 (12.4)	31.6 (11.5)	0.707
Mean count of individuals	2.62 (0.94)	2.70 (0.99)	2.57 (0.93)	0.671
Ward				0.469
2N	16 (40.0%)	8 (50.0%)	8 (33.3%)	
3O	24 (60.0%)	8 (50.0%)	16 (66.7%)	
Temperature (°C)	24.9 (1.06)	25.1 (0.89)	24.8 (1.16)	0.46
Relative humidity (%)	26.7 (3.17)	26.9 (2.64)	26.6 (3.53)	0.77
CO_2_ (ppm)	583 (47.9)	604 (56.9)	569 (35.6)	0.037 *

SARS-CoV-2 copies were detected by ddPCR using primers for the ORF1ab region. Door openings and the total number of individuals present in the corridor are the accumulated counts in the 1 h sampling period. Mean values for temperature, relative humidity, CO_2_ concentration, and people counting were calculated from the data recorded within 1 h sampling. Continuous variables with approximately normal distributions are presented as means ± standard deviations, and variables with skewed distributions are presented as medians (first and third quartiles). *: *p* < 0.05.

## Data Availability

Data collected in each air sampling is shown in Table 1.

## Abbreviations

The following abbreviations are used in this manuscript:

ASHRAE	American Society of Heating, Refrigeration and Air-Conditioning Engineers
CDC	Centers for Disease Control and Prevention
cDNA	Complementary DNA
CO_2_	Carbon dioxide
COVID-19	Coronavirus disease 19
ddPCR	Droplet digital PCR
DEPC	Diethyl pyrocarbonate
DNA	Deoxyribonucleic acid
dNTPs	Deoxynucleotide triphosphates
DTT	Dithiothreitol
dUTP	Deoxyuridine triphosphate
FAM	6-carboxyfluorescein
HFOT	High-flow oxygen therapy
HEX	Hexachlorofluorescein
HUSE	Hospital Universitari Son Espases
IRCU	Intermediate Respiratory Care Unit
NDIR	Non-dispersive infrared
OSHA	Occupational Safety and Health Administration
PCR	Polymerase Chain Reaction
qPCR	Quantitative polymerase chain reaction
REHVA	Federation of European Heating and Air Conditioning Associations
RNA	Ribonucleic acid
SARS-CoV-2	Severe acute respiratory syndrome coronavirus 2
WHO	World Health Organization
